# Case Report: Electroanatomic mapping as an early diagnostic tool in arrhythmogenic cardiomyopathy

**DOI:** 10.3389/fcvm.2024.1392186

**Published:** 2024-08-09

**Authors:** Jose F. de Melo, Samuel A. Shabtaie, Martin van Zyl, Jeremy D. Collins, Konstantinos C. Siontis

**Affiliations:** ^1^ Department of Internal Medicine, Mayo Clinic, Rochester, MN, United States; ^2^ Department of Cardiovascular Medicine, Mayo Clinic, Rochester, MN, United States; ^3^ Department of Radiology, Mayo Clinic, Rochester, MN, United States

**Keywords:** arrhythmogenic cardiomyopathy, electroanatomic mapping, premature ventricular contraction, task force criteria, case report

## Abstract

**Background:**

Abnormal substrate on invasive electroanatomic mapping (EAM) correlates with areas of myocardial thinning and fibrofatty replacement in Arrhythmogenic Cardiomyopathy (ACM). However, EAM parameters are absent from all sets of diagnostic criteria for ACM.

**Case summary:**

A 41-year-old female with no significant family history was referred for evaluation of frequent premature ventricular complexes (PVCs). Twelve-lead ECG showed diffuse low-voltage QRS complexes. Holter monitor showed 28% burden of PVCs with various morphologies consistent with right ventricular (RV) inflow and outflow tract exits. Transthoracic echocardiogram revealed normal biventricular function and dimension. Cardiac magnetic resonance revealed a mildly increased indexed RV end-diastolic volume with normal RV systolic function and no dyssynchrony, akinesia, dyskinesia, or late gadolinium enhancement. Electrophysiologic study demonstrated 2 predominant PVC morphologies that were targeted with ablation, in addition to extensive abnormality with low-voltage and fractionated electrograms in the peri-tricuspid and right ventricular outflow tract free wall regions with septal sparing, suggestive of RV cardiomyopathy. Subsequent genetic testing revealed two pathogenic variants in the desmoplakin and plakophilin-2 genes, confirming the diagnosis of ACM.

**Conclusion:**

Advanced RV electropathy can precede RV structural changes in ACM. Invasive evaluation of the electroanatomic substrate should be considered in select cases even when imaging findings are not diagnostic. Future iterations of ACM guidelines may need to consider EAM substrate as one of the diagnostic criteria. A high index of diagnostic suspicion for ACM should be maintained in patients with multifocal RV ectopy.

## Introduction

Arrhythmogenic cardiomyopathy (ACM) is an inherited condition caused by defects in the genes encoding cardiac desmosomes, characterized by fibrofatty replacement of the right ventricular (RV) myocardium (1), with biventricular and left-ventricular (LV) phenotypes being increasingly recognized (2). The disease typically manifests with ventricular tachyarrhythmias and RV systolic dysfunction (3). The first International Task Force diagnostic criteria for ACM was established in 1994. Subsequent revisions were introduced in 2010 and 2020 in an effort to improve sensitivity and increase earlier diagnosis of the disease (4).

It is well established that areas of abnormal substrate identified by invasive electroanatomic mapping (EAM) correlate anatomically with regions of myocardial thinning and fibrofatty replacement in ACM, and that the use of EAM may increase the sensitivity for detection of the disease (5, 6). However, EAM is absent from all sets of diagnostic criteria.

In this report, we present a patient without major structural cardiac abnormalities by multimodality imaging in whom abnormal findings by EAM led to the diagnosis of ACM with confirmation by genetic testing.

## Case presentation

**Figure 1:**
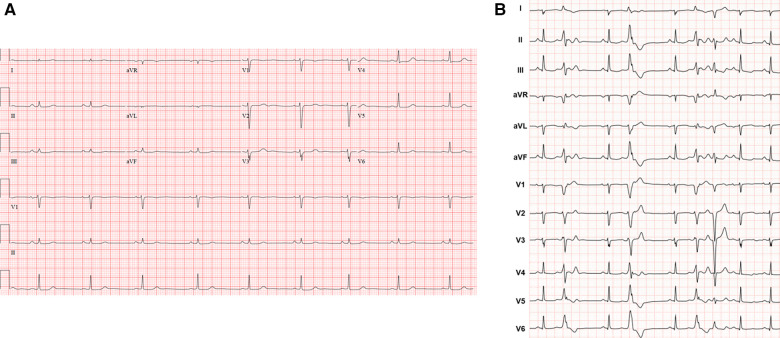
(**A**) Representative 12-lead electrocardiogram. (**B**) Twelve-lead Holter monitor tracing. Holter showed more than 5 different RV inflow and outflow tract PVC morphologies with a total burden of 28% over 24 h. Follow-up 24-h Holter 3 months post-ablation demonstrated 6% PVC burden.

**Figure 2:**
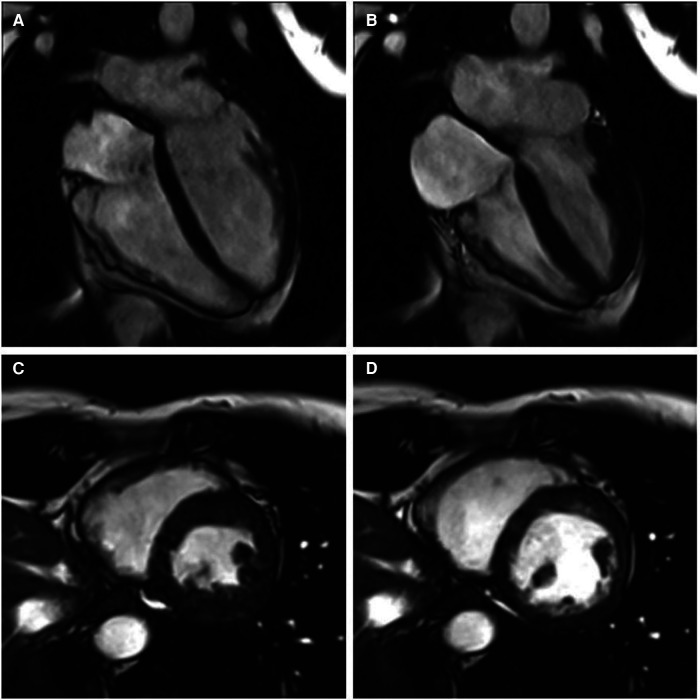
Cine cardiac magnetic resonance balanced steady state free precession cinegraphic imaging (**A**) 4-chamber end-diastole, (**B**) 4-chamber end-systole, (**C**) short axis end-diastole, and (**D**) short axis end-systole. Imaging demonstrated mild RV enlargement (RV end-diastolic volume index 107 ml/m^2^, normal range 51–103 ml/m^2^) without dyssynchrony, akinesia, or dyskinesia; normal biventricular function (RV EF 51%, LV EF 58%), and no late gadolinium enhancement or other structural abnormality.

A 41-year-old female with no significant past medical history and no family history of cardiomyopathy or sudden cardiac death was referred for evaluation of persistent frequent premature ventricular contractions (PVCs) following a catheter ablation performed at an outside institution. She was in her usual state of health until a year prior to evaluation, when she developed palpitations, dyspnea on exertion, chest pain, and pre-syncope. The initial evaluation at our institution revealed diffuse low voltage QRS complexes on the 12-lead electrocardiogram, but no inverted T waves or epsilon waves (Figure 1A). A 12-lead Holter monitor showed 28% burden (25,000/24 h) of PVCs with more than 5 different morphologies consistent with RV inflow and outflow tract (RVOT) exits (Figure 1B). A transthoracic echocardiogram revealed normal LV and RV ejection fraction, and normal biventricular dimensions. Cardiac magnetic resonance (CMR) imaging revealed a borderline increased RV chamber size (RV end-diastolic volume indexed for body surface area 107 ml/m^2^, normal range 51–103 ml/m^2^) with normal systolic function (RVEF 51%, LVEF 58%) without dyssynchrony, akinesia, or dyskinesia, and no findings suggestive of RV or LV fibrosis, myocarditis, infarction, or late gadolinium enhancement (Figure 2). There were no pertinent findings on physical examination with the exception of occasionally irregular heart sounds due to PVCs.

**Figure 3:**
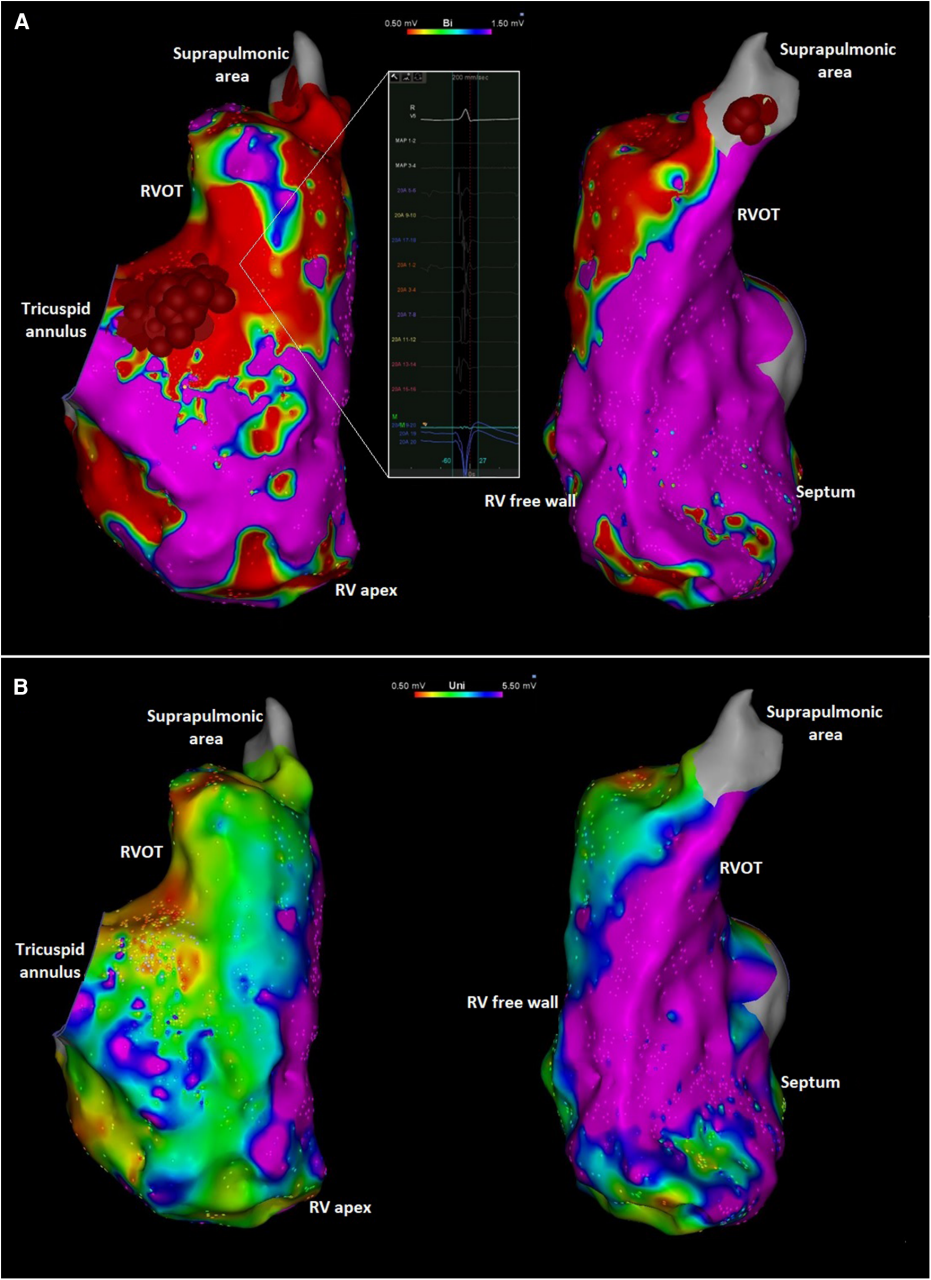
(**A**) Right anterior oblique (left) and left anterior oblique (right) views of the endocardial right ventricular bipolar electroanatomic maps showing low voltage along the tricuspid annulus and RVOT. The inlet shows an area with low amplitude and fractionated local electrograms. Ablation targets at the peritricuspid and suprapulmonic areas are also shown. The focal abnormality at the mid-distal septum likely reflects an area targeted during PVC ablation procedure previously performed at another institution. (**B**) Right anterior oblique (left) and left anterior oblique (right) views of the unipolar voltage mapping of the right ventricle showing low voltage in the peritricuspid area and RVOT. Red and purple colors represent low and normal voltage bipolar electrograms, respectively (bipolar: <0.5 mV and >1.5 mV; unipolar: <0.5 mV and >5.5 mV). Intermediate colors represent borderline voltage as shown in the scale in the top center of the figure.

An electrophysiologic study with catheter ablation was pursued. Endocardial high-density substrate bipolar voltage mapping with a PentaRay multielectrode catheter (Biosense Webster, Diamond Bar, CA) demonstrated extensive abnormality with low-voltage and fractionated electrograms on the free wall peri-tricuspid region and the RVOT free wall with sparing of the septum (Figure 3). Unipolar voltage mapping demonstrated similar area of low-voltage localized mainly to peritricuspid annulus and RVOT. The area of substrate abnormality was also verified with limited mapping with a contact force sensing catheter to ensure that the low voltage was not due to poor contact of the mapping catheter. There were no distinct late potentials and no inducible ventricular tachycardia (VT) with programmed ventricular stimulation with triple extrastimuli. Two predominant PVC morphologies were targeted with acute success at the anterior tricuspid annulus and the anteroseptal supra-pulmonic RVOT (Figure 3). These findings were overall suggestive of a RV-predominant cardiomyopathy despite the absence of definitive imaging findings.

Due to the EAM abnormalities, genetic testing for suspected ACM was recommended. The Invitae Arrhythmia and Comprehensive Cardiomyopathy panel identified two pathogenic variants in the desmoplakin (DSP, c.5028_5031del) and plakophilin-2 (PKP2, c.1912C>T) genes, confirming the underlying genetic cause for her presentation. Her estimated 5-year risk of sustained ventricular arrhythmias was 5.5% based on a recently developed risk stratification model (7). Additionally, because of the presence of two pathogenic ARVC-causing genetic variants, a primary prevention implantable cardioverter-defibrillator was recommended. At 1-year follow-up, the patient had improved symptoms and no interim sustained ventricular arrhythmias.

## Discussion

The criteria for the diagnosis of ACM have evolved for nearly three decades. Advances in cardiac imaging, mainly CMR, have allowed the recognition of varying disease phenotypes, which is reflected in the 2020 International Criteria wherein the diagnosis is heavily dependent on the identification of salient RV abnormalities by late gadolinium-enhanced CMR (8). However, the identification of fibrofatty infiltration by CMR, a hallmark characteristic of ACM, presents limitations. The spatial resolution of CMR might have insufficient sensitivity for detection of the structural changes in the early stages of the disease, particularly due to the thinness of the RV free wall (9).

In suspected ACM, invasive EAM can characterize the substrate abnormality corresponding to fibrofatty replacement in early stages of the disease and before these abnormalities are detected by imaging (6). The arrhythmogenic substrate of ACM is typically most pronounced in the epicardium, with endocardial abnormalities absent until later in the disease course (10). Epicardial mapping was not performed in our patient, but it is striking that endocardial EAM abnormality was present well before significant structural RV abnormalities. This is consistent with the notion that advanced RV electropathy can precede RV structural changes. This observation has significant implications in the diagnostic evaluation of ACM such that invasive evaluation of the electroanatomic substrate should be considered in select cases even when imaging findings are underwhelming. Additional benefit of EAM includes the ability to differentiate ACM from mimicking conditions such as myocarditis and cardiac sarcoidosis (11). Further, these patients often have additional indications for electrophysiologic procedures, including risk stratification with programmed ventricular stimulation or catheter ablation of ventricular arrhythmias, as was the case with our patient. The extent of bipolar low-voltage substrate abnormality has also been shown to correlate with noninvasive measures of disease severity (such as ECG and MRI-LGE abnormalities) (12) and may be an independent predictor of arrhythmic events such as sudden cardiac death, appropriate ICD intervention, and sustained VT (13).

The presence of frequent PVCs (>500 per 24 h) is a major diagnostic criterion for ACM (8). Differentiating idiopathic RVOT arrhythmias from those occurring in the setting of an ACM is critical given the marked differences in management and prognosis. The presence of multifocal ectopy with morphology characteristics of RV exit, as seen in our patient, should clue the clinician into the possibility of ACM.

Our case is particularly unique due to the presence of two pathogenic ACM mutations with PKP2 dominantly contributing to the RV abnormality and the DSP mutation contibuting to the LV abnormality, such as diffuse low voltage QRS. Our patient did not meet Modified Task Force or Padua criteria for ACM based on electrocardiographic, imaging, and genetic information available prior to invasive electrophysiologic testing. The value of EAM in strengthening the diagnosis of ACM has been demonstrated in patients who otherwise fulfilled Task Force criteria of ACM prior to invasive mapping (5, 6). Further, Corrado et al. reported abnormal RV EAM findings in a small subset of patients referred for RVOT VT ablation in the absence of echocardiographic evidence of RV myopathy (14). However, CMR imaging was used in only a few patients in that cohort. To our knowledge, the case presented herein is one of the first cases in the literature where invasive EAM findings in a patient with PVCs triggered subsequent genetic testing ultimately confirming the diagnosis of ACM. In another report, Castro et al. demonstrated the diagnosis of ACM in a patient who presented with RVOT VT initially considered to be idiopathic given normal CMR imaging (15). The patient underwent VT ablation where normal bipolar voltage was identified in the endocardium, but abnormal substrate was identified in the RV epicardium where the VT was successfully targeted. Subsequent genetic testing revealed a pathogenic PKP2 mutation leading to a definite ACM diagnosis. These cases add to accumulating evidence suggesting that invasive EAM is a valuable tool for the early detection of ACM.

**Figure 4:**
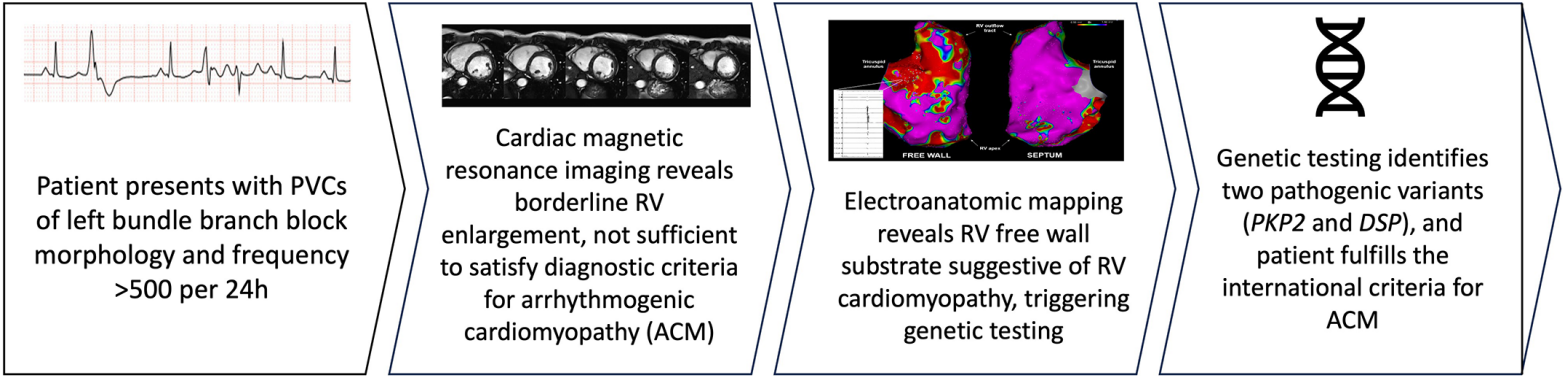
Summary figure.

In summary, the diagnosis of ACM can be challenging. Electrophysiologic substrate abnormalities can precede typical imaging-detected structural abnormalities (Figure 4). Abnormal RV substrate on invasive EAM mapping should be considered among the diagnostic criteria for ACM. A high index of diagnostic suspicion for ACM should be maintained in patients with multifocal RV ectopy.

## Data Availability

The original contributions presented in the study are included in the article/Supplementary Material, further inquiries can be directed to the corresponding author.
